# Screening for adulticidal bioactivity of South African plants against *Anopheles arabiensis*

**DOI:** 10.1186/1475-2875-10-233

**Published:** 2011-08-11

**Authors:** Rajendra Maharaj, Vinesh Maharaj, Neil R Crouch, Niresh Bhagwandin, Peter I Folb, Pamisha Pillay, Reshma Gayaram

**Affiliations:** 1 South African Medical Research Council, 491 Ridge Road, Overport, Durban 4001, South Africa; 2 Biosciences, CSIR, P.O.Box 395, Pretoria 0002, South Africa; 3 Ethnobotany Unit, South African National Biodiversity Institute, PO Box 52099, Berea Road 4007/School of Chemistry, University of KwaZulu-Natal, Howard College Campus, Durban 4041, South Africa; 4 South African Medical Research Council, P.O.Box19070, Tygerberg 7505, South Africa

## Abstract

**Background:**

This study was conducted to evaluate whether a selection of South African ethnomedicinal plants included in this study displayed insecticidal properties when screened against adult stages of the mosquito.

**Methods:**

381 crude extracts of 80 plant taxa in 42 families were sprayed onto ceramic tiles and screened using the cone bio-assay method for insecticide efficacy testing. Blood-fed, female *Anopheles arabiensis *mosquitoes were exposed to the treated tiles for a period of sixty minutes. Mosquito mortality was monitored for twenty-four hours.

**Results:**

Of all the extracts analysed, the highest activity was observed in *Ptaeroxylon obliquum *(Ptaeroxylaceae) and *Pittosporum viridiflorum *(Pittosporaceae), a single extract from each, exhibiting more than 50% mortality. A large proportion (81.63%) of the extracts tested displayed low levels of mosquitocidal activity. The remainder of the extracts (17.85%) exhibited no bioactivity (0% mortality).

**Conclusions:**

The screening results have shown that in accordance with WHO standards, none of the crude extracts tested had exhibited greater than 60% mortality against the adult stages of the malaria vector *Anopheles arabiensis*.

## Background

Even though there is adequate prevention measures and effective case management available, malaria remains one of the most important public health diseases resulting in approximately 300 million cases and an estimated 781 000 deaths annually [1]. Adult female anopheline mosquitoes have the ability to transmit malaria from an infected individual to a susceptible person. Vector control measures have, therefore, been established to control the transmission of the disease by targeting the carriers. Over the past few decades, the vector has however developed the ability to evade intervention measures, which target adult mosquitoes [2] thus exacerbating the problem for vector control programmes. The current vector control technique involves the use of residual insecticides which are sprayed onto walls and roofs of houses. This method, known as indoor residual house spraying (IRS) allows for a lethal dose of insecticide to adhere to the mosquito once it has rested on a sprayed surface [3].

Research conducted over the years has produced four main classes of chemically based insecticides: organochlorines, organophosphates, carbamates and pyrethroids [4]. Although these insecticides are known to be very effective, they continue to pose a potential health and environmental problem rendering them undesirable and arguably inappropriate for use in public health [5]. Furthermore, the continued long-term use of these chemicals has resulted in mosquitoes rapidly developing physiological resistance, which hinders vector control methods and leads to the recurrence of the disease. In the early 1950s the World Health Organization (WHO) eradication campaign introduced the large-scale use of DDT. However, during the subsequent decade pyrethroids replaced DDT in selected areas due to its low dose efficacy and opposition to its use by target communities. This change was short-lived as pyrethroids proved cost-ineffective relative to the health benefits derived. Resistance to both pyrethroids and DDT was subsequently observed in South Africa [6,7], challenging the notion that the rapid toxicological action of pyrethroids would significantly decrease the likelihood of this developing [8]. The inevitability of insecticide resistance therefore highlights the need for urgent development of additional adulticidal pesticides, since current products are predicted to become ineffective in the near future [9].

For years, research has been focused on finding insecticides of high efficacy, which are cost effective and environmentally safe. The use of indigenous plants has increasingly become the major avenue for research since these organisms contain an array of bioactive chemical compounds, some of which can be used to effectively kill or repel mosquitoes at various life stages. Furthermore, phytochemicals contained in specific plants may act as insecticides against both the aquatic (larvae) and adult stages of the mosquito. Alternatively, these chemicals can serve as vector growth inhibitors, preventing mosquito larval development [10].

Various studies have successfully isolated compounds from plants that display insecticidal properties. One such compound is rotenone, which is produced by species of *Derris *and *Lonchocarpus*, both of the Fabaceae or legume family [11]. Another commonly used natural insecticide, extracted from the flower heads of *Tanacetum cinerariifolium *(= *Chrysanthemum cinerariifolium*) (Asteraceae) is pyrethrum [12], which has been effective in insect pest control around the world. Due to its rich source of bioactive chemicals, the neem tree (*Azadarachta indica*) (Meliaceae) is one of the most significant and extensively researched of all medicinal plants [13]. Different parts of the tree have been used to treat a wide range of diseases in man and livestock as well as to eradicate disease vectors. Neem oil and extracts of neem seed kernels have also been found to be mosquitocidal [14]. Plants such as *Tagetes *(Asteraceae) species have been shown effective against the adult and immature stages of the mosquito, whilst *Eclipta paniculata *(Asteraceae) displayed significant larvicidal properties and *Polyalthia longifolia *(Annonaceae) exhibited both larvicidal and growth inhibition effects [15]. South Africa possesses a rich diversity of plant life with over 24,000 plant species, of which approximately 15% are ethnomedicinal (used traditionally for medicinal purposes) [16]. The importance of ethnomedicinal plants lies not only in their chemotherapeutic value in traditional health care but also in their potential as sources of biologically active entities. Several of the taxa selected for this study have earlier been studied for their anti-plasmodial properties [17] and a selection has been observed to provide some protection against mosquito bites [18]. This study was conducted to determine whether any indigenous or naturalized South African ethnomedicinal plants exhibit effective killing properties against adults of the malaria vector *Anopheles arabiensis*.

## Methods

### Selection and collection of plant material

A survey of relevant published literature, housed at the SANBI, on ethnomedicinal plants used in east and southern Africa revealed that a number of taxa have been reported to be used as mosquito repellents, or to repel or kill other invertebrates. However, given the limited quantity of documented data available, it was decided to not make a distinction between insect repellents and insecticides (both larva- and adulticides), but to rather consider a pool of plants with anti-insecticidal activity.

In order to select the most relevant taxa for screening, all were ranked following the application of weighted criteria, principally ethnobotanical and chemotaxonomic (including such elements as popularity in ethnomedicinal trade, reports on insecticidal and/or mosquitocidal application, reports on insect and/or mosquito repellent application, and the known presence and diversity of repellent/insecticidal constituents in the family to which it belongs). Higher weighting was provided to plants indigenous to the *Flora of southern Africa *region. A similar semi-quantitative selection method has previously been applied to identify and rank both anti-plasmodial [17] and mosquito repellent [19] plant candidates from South Africa. From the ranked list selected plants were collected throughout South Africa. Different plant parts, namely, leaves, root, stem, fruit, flowers, seeds, twigs and bark, and combinations of the above were sourced to generate extracts. In some instances, extracts were made of the whole plant. The plant organ(s) selected for extract preparation was based largely on availability at the time of collection.

The identity of plant material was determined at the National Herbarium of South Africa (PRE) where voucher specimens (cited in Additional File 1, Table S1) have been lodged.

### Extract preparation

Plant samples were separated into different components and dried in an oven at 30-60°C. The drying time and temperature varied depending on the nature of the plant part. Dried plant material was ground to a coarse powder using a hammer mill and stored at ambient temperature prior to extraction. For each extraction procedure, 100-500 g of powdered plant material was sequentially extracted, with cold dichloromethane (DCM), DCM/methanol (MeOH) (1:1), MeOH (CP Grade; Merck) and purified water. Organic extracts were concentrated by rotary vacuum evaporation below 45°C and then further dried *in vacuo *at ambient temperature for 24 h. The aqueous extracts were concentrated by freeze-drying. All dried extracts were stored at -20°C.

Carriers for the extracts, either acetone or distilled water, were used as the untreated negative control whilst deltametrin (K-Orthrine WG; Bayer), a known effective, water dispersable granule was used as the positive control. Crude plant samples were dissolved in either acetone (AR Grade; Merck) or distilled water depending on their initial extraction procedure, thus forming a 10 mg/ml solution. Dichloromethane and dichloromethane/methanol extracts were reconstituted in acetone whereas aqueous extracts were made up using distilled water.

### Insecticidal screening

A 1 ml volume of extract solution was sprayed onto a clean, dry, non-porous ceramic tile using a pre-calibrated Potter's Tower apparatus [20]. This instrument allowed for even application of precise amounts of test solutions to the tiles, following which they were air dried. Assays were initiated within 24 hours of spraying.

The assay methodology was conducted in accordance with WHO protocol [21] in which a standard bioassay cone was fixed over the sprayed tile and thirty, non-blood-fed, 2-5 day-old susceptible adult mosquitoes (*An. arabiensis*) were introduced into the cone. All bioassays were duplicated to ensure validity of results. The effect of the sample extract was measured by determining the knockdown rate, constituting temporary paralysis of the mosquitoes during the sixty-minute exposure period, and post-exposure mortality within twenty-four hours. In order to establish whether any plant extract warranted further investigation, stringent WHO criteria [21] was adapted for the screening of crude plant extracts. Therefore only crude extracts of plants inducing mortality greater than 60% were considered as potential insecticide candidates for further research and development.

## Results and Discussion

This study examined the mosquito adulticidal activity of a number of plants native to or naturalised in southern Africa, and included the testing of 381 crude plant extracts derived from 80 taxa in 42 families (Additional File 1, Table S1). To facilitate a thorough investigation of the plants assayed, extracts were produced from either the whole plant, and/or separate organs including fruit, flowers, leaves, stem, twigs, bark and root, when available. Results have been presented in the supplement document (Additional File 1, Table S1) alphabetically by family, genus and species, and thereafter as extracts in descending order of mortality (efficacy shown as the average of two replicates), twenty four hours post exposure. Despite strong anecdotal associations with malaria, a large number of the plants selected did not display any mosquitocidal activity.

The results of the investigation have shown that only two crude extracts (representing two taxa) exhibited more than 50% mortality, whereas 311 extracts induced mortality of between 1 and 49%. A total of 68 crude samples did not demonstrate any toxicity as indicated by a lack of mortality relative to the control.

To identify extracts with the greatest potential to yield insecticides, only samples inducing > 60% mortality after twenty fours exposure were considered for further analysis. However, the screening of crude, indigenous plant extracts revealed that none of the samples tested in the current study were effective at WHO standards against the malarial vector *An. arabiensis*.

Prior to the introduction of synthetic insecticides in vector control, many phytochemical insecticides, such as azadirachtin and turpentine amongst others, were used in the more developed countries [14]. With the advent of synthetic insecticides such as organochlorines and organophosphates, the search for botanical interventions was largely stalled until increasing insecticide resistance was detected. A review conducted by Shaalan *et al *[14] has found that there are a number of plant species worldwide that have the ability to cause acute and chronic toxic effects in mosquitoes, supporting the notion that new pesticides may yet be developed from plants.

Although communities are documented to traditionally use preparations of various plant organs as repellents through either topical application [18] or fumigation [22], no reports of plant extracts being used traditionally as mosquito adulticides have been found.

In some cases where the burning of specific plants as fumigants reportedly deters mosquitoes [22], laboratory-based assays have at times validated these traditional practises: leaves of *Corymbia citriodora *(Myrtaceae), and leaves and seeds of both *Ocimum kilimandscharicum *and *Ocimum suave *(Lamiaceae) had exhibited a significant repellent effect against *Anopheles gambiae *s.s and *An. arabiensis *during application of plant material by thermal expulsion [23]. The plant selection procedure employed in the current study grouped potentially insecticidal as well as potentially mosquito repellent species. Accordingly, some plants that have here shown non-promising mosquito adulticidal activity might exhibit repellent activity when used as fumigants.

In the current study, the plants producing the highest mortality were *Ptaeroxylon obliquum *(Ptaeroxtlaceae) and *Pittosporum viridiflorum *(Pittosporaceae). Previous studies have shown that both these trees are known to have medicinal properties [24], indicative of bioactivity. The study also revealed that extracts of different parts of the same species were differentially active. *Ptaeroxylon obliquum *leaf extracts induced 57% mortality of adult *An. arabiensis*, whereas the stem gave 33% and the roots only 27%. This may indicate that the phytochemicals of interest may be localized in and/or concentrated in particular plant organs. This investigation looked preferentially at roots, stems and leaves and was guided by the selection criteria used in choosing the plant species.

Although representatives from the Ptaeroxylaceae and Pittosporaceae yielded the best overall results, most species inducing > 25% mortality belonged to the Asteraceae.

In addition to the relation of adulticidal activity to the part of plant tested, differential tolerances of mosquitoes to extract type was observed following use of four different solvents in their preparation. For the aqueous extract of *Ptaeroxylon obliquum*, leaves revealed 57% activity when tested whereas the organic extract of the same plant part yielded 7% activity. Similar observations were described in another study [25] substantiating the notion that different solvents used during the extraction process can influence the toxicity of the plant extract on the mosquito species. Similarly, another study [26] found that the method of extraction affected the acaricidal constituents of plant extracts, both quantitatively and qualitatively.

## Conclusions

Overall, the results show that 82% of the plants investigated displayed low levels of adulticidal activity. In terms of effectiveness, none of these extracts were significantly active compared to the positive controls, based on WHO standards. Further screening and bioassay-guided fractionation of these extracts was, therefore, not pursued. Although this research has been conducted on *Anopheles arabiensis *(the principal vector of malaria in South Africa), the observed activities may differ for other local, non-vector, nuisance mosquito species, or for other virulent vectors found elsewhere in malaria endemic regions of the globe.

This extensive, albeit pioneering study of the mosquito adulticidal properties of selected South African flora has shown that according to WHO standards, none of the plants screened possessed insecticidal properties against the malaria vector, *An. arabiensis*. Further work on different chemotypes of the two most promising candidates *P. obliquum *and *P. viridiflorum*, and their closest relatives, could yet result in the selection of more research-worthy insecticidal candidates from the southern African region.

## Competing interests

The authors declare that they have no competing interests.

## Authors' contributions

RM was involved in the design of the study and supervising the bioassays. RG carried out the experiments and was involved in the interpretation of the results. NRC rationally selected suitable plant candidates for investigation. VM and PP were responsible for the extract preparation. NB and PF had coordinated and provided scientific inputs into the entire study. All authors read and approved the final manuscript.

## Supplementary Material

Additional file 1 **Table 1 Mosquito adulticidal screening results of extracts of South African ethnomedicinal plants**. Click here for file
